# Additive Effects of Threat-of-Shock and Picture Valence on Startle Reflex Modulation

**DOI:** 10.1371/journal.pone.0054003

**Published:** 2013-01-16

**Authors:** Florian Bublatzky, Pedro M. Guerra, M. Carmen Pastor, Harald T. Schupp, Jaime Vila

**Affiliations:** 1 Department of Psychology, School of Social Sciences, University of Mannheim, Mannheim, Germany; 2 Department of Psychology, General Psychology, University of Konstanz, Konstanz, Germany; 3 Department of Personality, University of Granada, Granada, Spain; 4 Department of Psychology, Jaume I University, Castellón, Spain; University of Gent, Belgium

## Abstract

The present study examined the effects of sustained anticipatory anxiety on the affective modulation of the eyeblink startle reflex. Towards this end, pleasant, neutral and unpleasant pictures were presented as a continuous stream during alternating threat-of-shock and safety periods, which were cued by colored picture frames. Orbicularis-EMG to auditory startle probes and electrodermal activity were recorded. Previous findings regarding affective picture valence and threat-of-shock modulation were replicated. Of main interest, anticipating aversive events and viewing affective pictures additively modulated defensive activation. Specifically, despite overall potentiated startle blink magnitude in threat-of-shock conditions, the startle reflex remained sensitive to hedonic picture valence. Finally, skin conductance level revealed sustained sympathetic activation throughout the entire experiment during threat- compared to safety-periods. Overall, defensive activation by physical threat appears to operate independently from reflex modulation by picture media. The present data confirms the importance of simultaneously manipulating phasic-fear and sustained-anxiety in studying both normal and abnormal anxiety.

## Introduction

A large body of evidence supports the notion that the startle reflex is modulated by defensive system activation. When anticipating danger, the startle reflex is potentiated as compared to control conditions, for instance in aversive conditioning paradigms [1] or when participants are verbally instructed that they might receive an electric shock during sustained threat periods [2]. Furthermore, during passive picture viewing, the startle reflex is potentiated for unpleasant images, and inhibited for pleasant contents [3]. These results have been interpreted from the perspective of motivational priming, assuming that defensive activation primes defensive reflexes such as the startle response, which are conversely inhibited during appetitive motivational system activation. As defensive activation is a key component in fear and anxiety, considering the preceding conditions (e.g. phasic or sustained cues) is important for the understanding of both normal and abnormal processes.

Learning about aversive events is critical in organizing defensive behavior. Accordingly, the mere verbal instruction about potential threats is sufficient to prime defensive response programs [2], [4] and facilitates the processing of sensory information [5]–[8]. However, only few studies addressed the nexus of aversive contingencies in mediating fear and anxiety learning by means of different cue types. Recent research has begun to explore the interaction of anticipatory anxiety and emotional picture processing. Data from clinical population suggest differences in the neural organization of anticipatory anxiety and emotional picture processing. For instance, startle modulation prompted by instructed threat-of-shock was impaired in patients with left rather than right unilateral temporal lobectomy, whereas the opposite pattern was observed when participants viewed emotional pictures [9]. Furthermore, a recent study examined startle reflexes in the context of pleasant and unpleasant pictures signaling either threat-of-shock or safety [4]. When pleasant pictures served as threat cues, startle reflex was potentiated as compared to safety condition. In contrast, for unpleasant pictures, blink magnitude did not differ between threat-of-shock or safety conditions. Thus, modulation of the startle reflex was sensitive to the valence of cues signaling imminent danger. Measuring event-related potentials, a further study investigated the interaction of threat-of-shock and affective picture processing, when both manipulations coincided but the pictures were unrelated to threat/safety conditions [5]. Revealing a valence-specific effect of anticipatory anxiety on affective picture viewing, facilitated processing of pleasant cues was observed during threat-of-shock compared to safety conditions.

Building upon these findings, the present study examined coincident effects of sustained periods of anticipatory anxiety (72 s) and emotional pictures presented as a continuous stream (4 s), when both manipulations were unrelated. Measuring startle reflexes, the main purpose was to explore whether the concurrent activation of motivational systems by phasic picture cues and sustained periods of unpredictable threat-of-shock operate simultaneously but independent from each other, or whether they exert synergistic effects [4]–[6]. As an additional measure of defensive activation, electrodermal activity was expected to be increased during threat-of-shock compared to safety periods [10].

## Methods

### Participants

Participants were 36 healthy volunteers (12 males) between the ages of 18 to 27 (*M* = 22) recruited from University of Granada. Because of excessive noise in orbicularis oculi EMG, 3 participants (1 male) were excluded from startle data analyses.

All participants provided written informed consent to the study protocol, approved by the Ethic Review Board of the University of Granada and in accordance with the declaration of Helsinki.

### Materials and Design

Fifty-four pictures were selected from the International Affective Picture System (IAPS) [11] depicting people either in neutral (e.g. non-emotional situations), pleasant (e.g., erotica) or unpleasant situations (e.g. mutilation). IAPS numbers of the pictures used in the current study are: Pleasant, 4141, 4180, 4232, 4235, 4290, 4460, 4490, 4530, 4538, 4550, 4606, 4611, 4653, 4658, 4670, 4680, 4690, 4694; Neutral, 2102, 2104, 2191, 2305, 2358, 2372, 2383, 2396, 2397, 2435, 2495, 2513, 2515, 2560, 2570, 2580, 2850, 5410; Unpleasant, 3010, 3015, 3061, 3063, 3064, 3102, 3110, 3120, 3130, 3500, 3530, 6250, 6313, 6315, 6350, 6510, 6550, 6570.

Highly arousing emotional picture contents were selected, as these materials elicit most pronounced modulations in defensive reflex, autonomic measures, and brain imaging studies [12]–[14]. Categories differed in terms of normative valence and arousal ratings (pleasant *M* = 6.3 and 5.8, neutral *M* = 5.4 and 3.3, unpleasant *M* = 1.9 and 6.6), *Fs*(2,34) = 271.35 and 154.49, *ps* <.001. All post hoc comparisons were significant, *ps* <.01.

**Figure 1:**
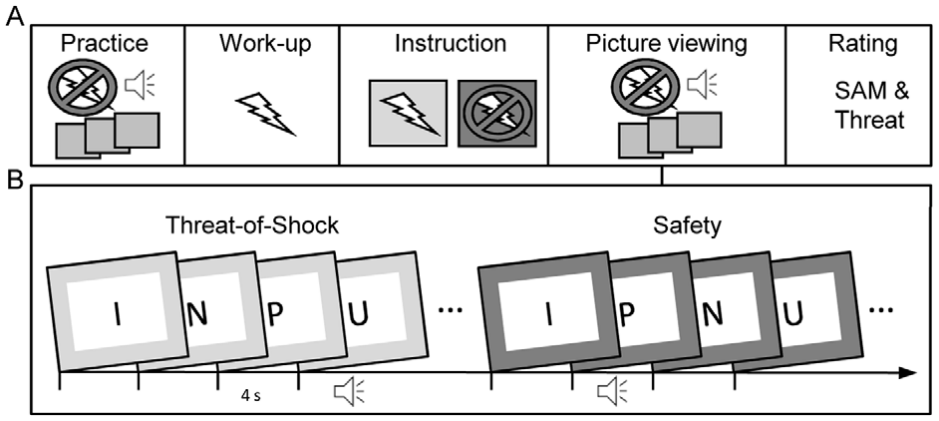
Schematic illustration of the experimental procedure (A) and stimulus presentation (B). Emotional and neutral pictures were randomly presented in a continuous picture stream (each 4 s). Participants were verbally instructed that the colored picture frames (blue or green) indicated either threat-of-shock or safety periods. Abbreviations P, N, U refer to pleasant, neutral, and unpleasant picture contents; I refers to instructions slides announcing “Shock possible” or “No shock” preceding each 72-s period of threat-of-shock or safety.

The IAPS pictures (640×480 pixels) were fleetingly presented for 4 s without perceivable inter-stimulus interval (see Figure 1). IAPS pictures were presented in random order with no more than three repetitions of the same picture category and the picture set was repeated four times. Surrounding the pictures, two colored background frames (blue/green; 1024×768 pixels) signaled experimental conditions of threat-of-shock or safety. Participants were verbally instructed that one specific frame color (e.g. blue) indicated the possibility to receive electric shocks (“threat condition”), whereas the other color frame (e.g. green) signaled the “safety condition.” Threat/safety signals were presented continuously alternating in 12 blocks of 18 pictures (6 pleasant, 6 neutral, 6 unpleasant randomly presented within each block). Corresponding instruction slides (5 s) preceded each threat/safety condition in order to help participants to follow up the procedure. Color assignment to conditions and block order (first block threat/safe) were counterbalanced across participants.

Startle responses were provoked by 105 dB, 50 ms white noise with instantaneous rise time, produced by Coulbourn V85–05 noise generator (Coulbourn Instruments, Whitehall, PA), gated through IMG Stage-Line® amplifier and presented over matched PPA-1 headphones. Probes were delivered 1, 1.5 or 2 s after picture onset in 72 trials, equally often for each picture category within each block (15.4 s mean distance between startle probes).

Pictures were presented on a 22-inch monitor located 1 m in front of the participants. Electrical pulses (max. 2.2 mA, 100 ms) were generated by a Letica-shock-module (Letica, Barcelona, Spain), and administered to the left forearm during shock-workup procedure. Stimulus control and physiological data acquisition were accomplished using VPM [15] and Presentation software (Neurobehavioral Systems, Inc., Albany, USA).

### Data Recording and Reduction

Eyeblinks were recorded electromyographically from the orbicularis oculi muscle with Ag/AgCl-electrodes. Raw EMG signals were amplified (5K) and bandpass filtered (13–1000 Hz), using Coulbourn V75–04 bioamplifier, then rectified and integrated on-line using Coulbourn V76–23 module (time constant 20 ms). The sampling rate for the integrated signal was 1000 Hz, recorded from 50 ms prior to 300 ms after probe onset. A computer program scored startle blink magnitude peak and onset latency interactively controlled while blinded to the conditions [16]. Raw values were standardized across probe trials, within individuals, and transformed to T-scores ((magnitude - mean magnitude)/SD) * 10+50).

Skin conductance activity was recorded through Ag/AgCl-electrodes, placed on the hypothenar eminence of the left palm, using Coulbourn V71–23 module with a sampling rate of 20 Hz. Averages were computed off-line for each 4 s picture period and tonic changes were determined by subtracting activity in 3-s before the first picture onset (within each block) from the means across periods. Logarithms of raw scores (log(change+10)) were computed for statistical analyses for non-startle trials only (i.e. to clean SCL from responses to startle probes, probe trials and the first following trial were excluded). To parallel a previous study utilizing ERP measures [5], pictures were presented in continuous sequences (no ITIs). Accordingly, phasic skin conductance changes to picture cues were affected by the prestimulus level and therefore not reported here. However, as picture categories were equally distributed within and across threat/safety periods, block wise analyses of the SCL data remained unaffected by picture content.

### Procedure

After sensors were attached, 12 practice trials were presented, including pictures, frames and two initial startle probes (excluded from analyses). Then the shock electrode was placed and a brief workup procedure was carried out to ensure credibility of the threat-of-shock instruction. In order to set the shock intensity individually at a level rated as “maximal unpleasant but not painful” participants received up to ten shocks with increasing intensity preceding the experiment [5], [6]. Participants were then told that the intensity of the electric shocks given during the experiment would be equal to the most unpleasant test stimulus. Afterward, main instructions regarding which color frame signaled threat-of-shock or safety conditions were given. Besides, participants’ task was to passively view all presented pictures. During the experiment, no shocks were administered. This was to avoid sensitization effects associated with shock delivery [17], and because sustained and robust threat-of-shock effects can be produced by mere verbal instructions [2], [4]–[6], [18]. At the end of the experiment, participants rated hedonic valence and arousal of threat/safety conditions using the Self-Assessment-Manikin and a debriefing interview was completed.

### Data Analysis

Separate *t*-tests for valence and arousal ratings were conducted on self-reports of threat and safety conditions.

To assess combined effects of threat-of-shock and picture valence on the startle reflex, repeated measures ANOVAs including the factors Picture Category (pleasant, neutral, unpleasant) and Condition (threat-of-shock, safety) were performed. Furthermore, to examine the time course of threat-of-shock and affective picture modulation, an additional factor (Time) was included by averaging the beginning, middle and last part of the experiment (i.e., 2 blocks per condition, 4 probed trials for each picture category). The resulting statistical design was Picture Category (3)×Condition (2)×Time (3).

Skin conductance changes were analyzed with repeated measures ANOVAs, including the factors Condition (2) and Time (3) summarizing the beginning, middle and last part of the experiment (i.e., 2 blocks each, averaging 15 to 19 trials in total). Greenhouse-Geisser corrections were applied where relevant.

## Results

### Self-report Data

Participants perceived threat-of-shock periods as more unpleasant (*M* = 2.83, *SD* = 1.2) than safety periods (*M* = 5.86, *SD* = 1.4), *t*(35) = −8.81, *p*<.001. In addition, threat-of-shock condition was rated as more arousing (*M* = 6.78, *SD* = 1.5) than safety condition (*M* = 3.92, *SD* = 1.9), *t*(35) = 7.44, *p*<.001.

### Startle Reflex

#### Threat-of-shock and picture category

Main effects regarding threat-of-shock and picture valence were replicated. Startle response magnitude was significantly increased during threat-of-shock as compared to safety conditions, F(1,32) = 65.76, p<.001. In addition, startle reflex was modulated by picture valence, F(2,64) = 8.13, p<.001, ε = .98. Post-hoc tests revealed that blink magnitude for unpleasant pictures was potentiated as compared to pleasant pictures, F(1,32) = 17.38, p<.001, and larger but not significantly different from neutral pictures, F(1,32) = 2.08, p = .16. Moreover, blink magnitude for pleasant pictures was inhibited in contrast to neutral pictures, F(1,32) = 6.33, p<.05.

**Figure 2:**
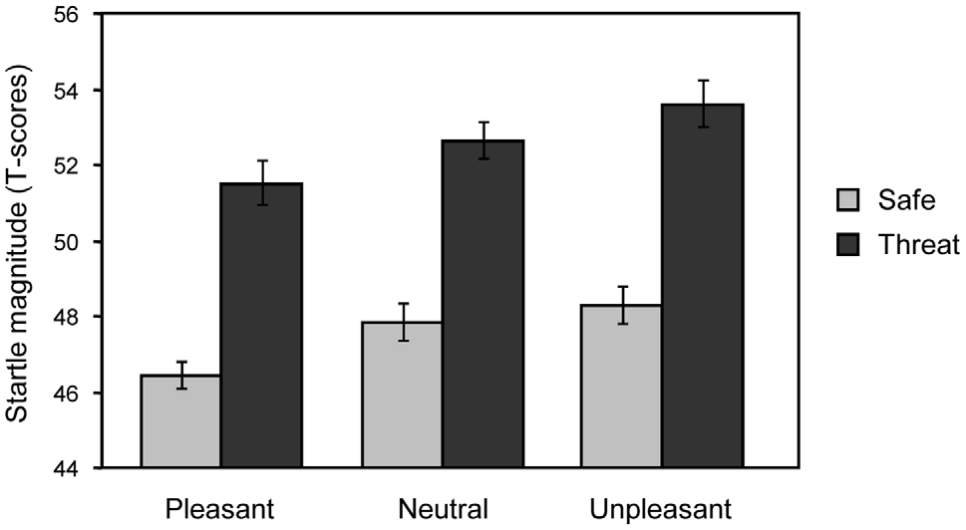
Mean magnitude (±SEM) of startle reflex as a function of threat-of-shock or safety, for pleasant, neutral and unpleasant pictures.

Of main interest, the interaction of hedonic picture valence and instructed threat condition was not significant, *F* <1 (see Figure 2). Exploratory post-hoc comparisons between threat and safety conditions showed that startle response was similarly potentiated for each picture category, *Fs*(1,32) >31.92, *ps* <.001. Interestingly, threat-of-shock potentiated startle for both pleasant and neutral pictures exceeded significantly the startle potentiation for unpleasant pictures in safety condition, *Fs*(1,32) >12.88, *ps* <.01. Additionally, startle modulation by picture valence was present in both safety and threat-of-shock conditions, *Fs*(2,64) >3.37, *ps* <.05, ε = .91 and.99.

#### Modulation over time

Including the additional factor Time revealed that neither the three-way interaction Category×Condition×Time nor the Category×Condition interaction (tested separately for each time window) approached significance, all *Fs*<1.

As expected, startle blink magnitude decreased along the experiment due to habituation, *F*(2,62) = 323.58, *p*<.001, ε = .95. Furthermore, threat-of-shock and picture valence effects varied across time, Time×Condition *F*(2,62) = 13.24, *p*<.001, ε = .81, Time×Picture Category, *F*(4,124) = 3.4, *p*<.05, ε = .67. Threat-of-shock effects were pronounced during the first, *F*(1,31) = 45.58, *p*<.001, and second time period, *F*(1,32) = 53.76, *p*<.001, while still significant in the third time period, *F*(1,32) = 14.19, *p*<.001. Picture category effects were also reduced across time. Specifically, affective modulation of blink magnitude was significant at the first, *F*(2,64) = 4.8, *p*<.05, ε = .91, second, *F*(2,64) = 10.6, *p*<.001, ε = .98, but not the third time period, *F*(2,64) = 1.28, *p* = .28, ε = .99.

### Skin Conductance Level

**Figure 3:**
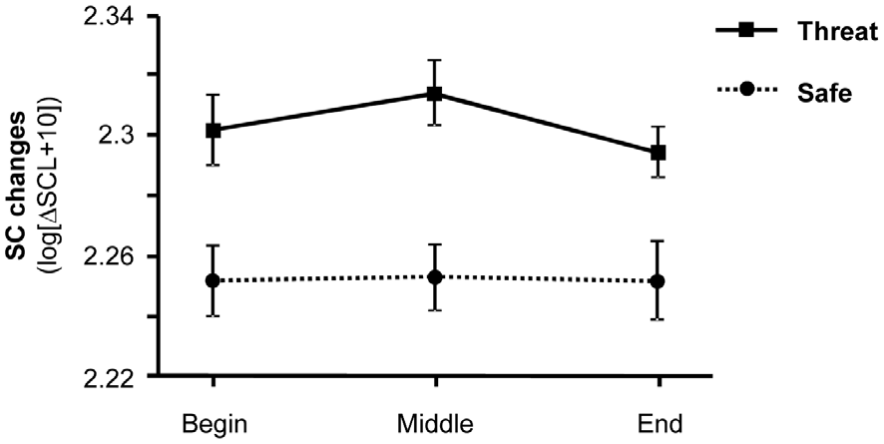
Mean skin conductance level (±SEM) for threat-of-shock and safety conditions across time course (begin, middle, end) of the experiment.

Tonic electrodermal changes were enhanced during threat-of-shock compared to safety conditions, *F*(1,35) = 14.45, *p*<.001 (see Figure 3). This differentiation was sustained along the entire experiment, as indicated by a non-significant interaction of Condition and Time, *F* <1, and main effect of Time, *F* <1. Exploratory follow-up analyses revealed greater skin conductance level for threat-of-shock as compared to safety periods in the first, *F*(1,35) = 6.01, *p*<.05, second, *F*(1,35) = 13.07, *p*<.001, and third time period of the experiment, *F*(1,32) = 6.62, *p*<.05.

A supplementary analysis of the electrodermal data for the excluded trials (containing startle probes and the first following trial) revealed augmented skin conductance for threat as compared to safety periods, *F*(1,35) = 14.35, *p*<.001. Skin conductance decreased over Time, *F*(2,70) = 6.74, *p*<.01, but there was no interaction of Condition×Time, *F* <1.

## Discussion

The present study examined the modulation of the eyeblink startle reflex as a function of sustained anticipatory anxiety and affective picture valence. Key findings regarding both manipulations were replicated. Specifically, the startle blink magnitude was potentiated when anticipating a rather unpredictable aversive event [2], and unpleasant pictures potentiated the startle reflex compared to pleasant pictures during safety periods [3]. The novel finding was that anticipatory anxiety and picture valence exhibited additive effects on the startle reflex modulation. Specifically, affective startle modulation remained present even at the pronounced startle potentiation level due to threat-of-shock instructions. Thus, defensive activation by unpredictable physical threat (anxiety) seemed to operate independently from reflex modulation by picture media (phasic fear).

Investigating threat-of-shock and emotional picture processing, Bradley and colleagues [4] found a significant interaction of both variables. In this study, pleasant and unpleasant pictures served as cues for threat/safety. Startle potentiation was observed when pleasant but not unpleasant pictures signaled threat-of-shock compared to safety. The finding that pleasant pictures no longer inhibited startle reflexes when becoming a signal of imminent danger demonstrates the flexible and rapid adjustment according to environmental contingencies. Here, a markedly different pattern was observed when pleasant, neutral and unpleasant pictures were presented simultaneously but unrelated to the contextual threat/safety signals, as blink magnitude was inhibited compared to unpleasant images in each condition. Thus, the interaction of anticipatory anxiety and hedonic picture valence may critically depend on whether pictures are predictive of imminent danger (cf. [4], [6]) or unrelated to the threat-of-shock manipulation as in the current design.

The finding of an additive relationship between threat-of-shock and picture valence may be specific to motor output stages. Similar to the current design, a recent study examined the perceptual and evaluative processing of emotional pictures by measuring event-related potentials [5]. A significant interaction of threat-of-shock and picture valence was observed. Threat-of-shock compared to safety conditions specifically affected pleasant picture processing, which elicited a sustained negative difference potential over occipital regions in a 80–580 ms time window. Thus, pleasant stimuli mismatching the current state of anticipatory anxiety may draw more attentional resources during stimulus encoding. Accordingly, the relationship between threat-of-shock and picture valence may vary across response measures indexing processing priorities on the perceptual/evaluative (e.g. fast information intake, mismatch detection) and motor response stage (e.g. defensive activation to respond to potential threats) [19]. This hypothesis is consistent with research measuring various responses elicited by startle probes during emotional picture processing [20]–[24]. Whereas the P3 to startle probes may index greater attention allocation to affective pictures, the reflexive eyeblink is modulated by sequential and sometimes concurrent processes (e.g. attentional inhibition and affective modulation [20]). Overall, the simultaneous measurement of motivational response priming (e.g. blink reflex) and allocation of attentional resources (probe P3) appears as promising tool in future studies to examine the relationship of anticipatory anxiety and emotional picture processing.

Both sustained threat-of-shock periods and phasic processing of unpleasant pictures elicited potentiated startle reflexes in the current study. Thus, the startle reflex remained sensitive to picture valence even in a threatening context. Several findings support the notion that threat-of-shock is more powerful in activating the human defense system compared to emotional picture media [25]. As illustrated in Figure 2, startle potentiation associated with threat-of-shock was larger compared to effects mediated by emotional picture processing. Furthermore, startle potentiation was more sustained across time for threat-of-shock as compared to picture valence effects. Finally, similar to previous research [4], [10], threat-of-shock elicited enhanced electrodermal activity, which was sustained throughout the whole experiment. In activating the defense system, the relative greater effectiveness of the threat-of-shock manipulation compared to symbolic picture media is presumed to reflect the real-life imminence of physical danger [26], which can occur at unpredictable times [27]. However, despite pronounced differences in anticipatory anxiety reflected in overall startle magnitude, this reflex faithfully responded to picture valence similar in terms of magnitude and reliability as during safety conditions. Accordingly, the lack of interactivity between threat-of-shock and unpleasant picture content may rather reflect experimental settings (i.e. picture content not predictive for electric shocks) than possible ceiling effects (cf. [1], [4]). More likely, the present results may refer to an arousal-based impact of aversive anticipation on defensive reactivity. For instance, recent research found startle potentiation while anticipating emotionally arousing pictures (both pleasant and unpleasant), in contrast to neutral stimuli [28]–[31]. Analogously, the anticipation of aversive events while viewing task irrelevant pictures might reflect emotional intensity rather than the hedonic valence of the anticipated event.

These findings may be interpreted from the perspective of the defense cascade model [3]. In analogy to the predator imminence in animal research [26], physiological responses seem to change sequentially depending on the motivational impact (e.g. distance of threat) of the approaching event. For instance, Löw and colleagues [8] obtained similar physiological mobilization patterns during looming appetitive (monetary reward) and aversive (threat-of-loss) outcomes. Correspondingly, the anticipation of aversive events while viewing task-irrelevant pictures might reflect emotional arousal (enhanced SCL during threat-of-shock) rather than the hedonic valence of the anticipated event. However, future studies would need to detail autonomic responses to phasic stimuli in the presence of sustained potential threats. Notwithstanding, the present startle data support the notion of highly flexible motivational systems that dynamically adjust to affective foreground and contextual conditions.

The possibility to assess both aversive anticipation and emotional picture effects simultaneously may be informative in the study of the anxiety disorder spectrum [1], [32]–[35]. For instance, there is broad evidence for a differentiation between phasic fear and anxiety in animals regarding their behavioral, anatomical, and functional underpinnings [34]. The same model seems to apply to humans [35], [36]. Understanding the differential effects of both kinds of defensive behaviors would contribute to elucidate the mechanisms underlying anxiety disorders (e.g. generalized anxiety disorders) as opposed to those underlying fear (e.g. specific phobia). Furthermore, the threat-of-shock paradigm may contribute to the understanding of extinction processes. Extending previous research [2], [5], verbally mediated threat contingencies hold effective in activating the defense system, even without reinforcement as in the present design. Thus, an important follow-up question refers to the stability of anticipatory anxiety effects.

Several limitations of the present study need to be acknowledged and may be addressed in future research. First, the temporal design features (4 s picture presentation, no inter-trial interval) prevented the direct analysis of the interaction of threat-of-shock and picture valence by means of skin conductance data. Accordingly, accounting for the present hypothesis of concurrent but independent activation of motivational systems by fleeting picture cues and sustained threat signals, additional measures of phasic sympathetic activation (e.g., skin conductance responses to affective stimuli and startle probes) would be needed. Finally, to elucidate effects of habituation (picture repetition) and extinction (threat repetition) on autonomic and reflex activity the usage of block designs (e.g. blocked presentation of pictures with the same hedonic valence) and threat/safety periods varying in predictability would be highly informative [27], [37]–[40].

In summary, anticipating an aversive event and passively viewing unpleasant pictures potentiated the startle reflex magnitude. When these two avenues that activate the human defense system – threat-of-shock and emotional picture media – coincide but have no inherent relationship, the startle reflex is sensitive to both manipulations. Despite similar and presumably shared neural structures and pathways [39], both manipulations exhibited additive effects in the present study. Awaiting further empirical tests, the present startle data provide no support for the notion that defensive activation by anticipatory anxiety sensitizes the processing of unrelated aversive cues. Whether independent effects of anticipation of real events and emotional picture processing are also observed for pleasant stimuli [8] needs to be determined in future research.
